# Experimental Evidence of the Benefits of Acupuncture for Alzheimer's Disease: An Updated Review

**DOI:** 10.3389/fnins.2020.549772

**Published:** 2020-12-21

**Authors:** Chao-Chao Yu, Yan-Jun Du, Shu-Qin Wang, Le-Bin Liu, Feng Shen, Li Wang, Yuan-Fang Lin, Li-Hong Kong

**Affiliations:** ^1^Department of Tuina, Shenzhen Traditional Chinese Medicine Hospital, Shenzhen, China; ^2^The Fourth Clinical College of Guangzhou University of Chinese Medicine, Shenzhen, China; ^3^College of Acupuncture and Orthopedics, Hubei University of Chinese Medicine, Wuhan, China; ^4^Department of Rehabilitation Medicine, Hubei Rongjun Hospital, Wuhan, China

**Keywords:** acupuncture, Alzheimer's disease, experimental evidence, review, mechanisms

## Abstract

As the global population ages, the prevalence of Alzheimer's disease (AD), the most common form of dementia, is also increasing. At present, there are no widely recognized drugs able to ameliorate the cognitive dysfunction caused by AD. The failure of several promising clinical trials in recent years has highlighted the urgent need for novel strategies to both prevent and treat AD. Notably, a growing body of literature supports the efficacy of acupuncture for AD. In this review, we summarize the previously reported mechanisms of acupuncture's beneficial effects in AD, including the ability of acupuncture to modulate Aβ metabolism, tau phosphorylation, neurotransmitters, neurogenesis, synapse and neuron function, autophagy, neuronal apoptosis, neuroinflammation, cerebral glucose metabolism, and brain responses. Taken together, these findings suggest that acupuncture provides therapeutic effects for AD.

## Introduction

Alzheimer's disease (AD) is the most common neurodegenerative disease among elderly populations and accounts for nearly 80% of all dementia diseases. AD is characterized by progressive memory decline, executive dysfunction, personality and behavioral changes, and other neuropsychiatric syndromes (McKhann et al., 2011). The pathological hallmark of AD is extracellular senile plaque deposition containing Aβ and intracellular neurofibrillary tangles (NFTs) composed of hyperphosphorylated tau proteins (Hane et al., 2017). However, the pathogenesis of AD is complicated and remains largely unclear. It is widely accepted that the occurrence of AD is closely related to aging (Hou et al., 2019). As the global population ages, the morbidity of AD is thus also increasing. As a result, it is estimated that by 2050, there will be 138 million people with AD worldwide, posing a tremendous challenge to global healthcare (Alzheimer's, 2015). Although acetylcholinesterase inhibitors have been approved by the US FDA for the treatment of AD, their efficacy at improving cognitive function and preventing AD progression is less than satisfactory (Mohammad et al., 2017). Sodium oligomannate (GV-971), a marine algae-derived oral oligosaccharide, able to recondition the gut microbiota and alleviate neuroinflammation (Wang et al., 2019), was recently approved in China for the treatment of mild to moderate AD (Syed, 2020). However, more experimental and clinical evidence is needed regarding the mechanism of action, long-term efficacy, and safety of sodium oligomannate. Therefore, there remains a great clinical need for effective strategies for preventing and treating AD.

Acupuncture, a crucial practice in traditional Chinese medicine, is one of the most popular complementary and alternative therapies and is accepted by the World Health Organization and National Institutes of Health. Acupuncture is a relatively safe procedure in which stainless steel needles are inserted into acupoints to achieve the sensation of *deqi* and produce therapeutic effects. The effects of acupuncture can be further enhanced by electrical stimulation or manual manipulation. In electroacupuncture (EA), electrical stimulation is applied via acupuncture needles at a certain current and frequency accurately. In China, acupuncture has a long history of use in the treatment of neurological diseases. Mounting evidence supports that acupuncture provides satisfactory effects for various neuropsychiatric disorders including vascular dementia (Yu et al., 2006; Xiao et al., 2018), depression (Wang et al., 2016), and insomnia (Yin et al., 2017). Notably, there is also accumulating clinical and experimental evidence for acupuncture as a potential treatment for AD. Several systematic reviews and meta-analyses have concluded that acupuncture alone (Huang et al., 2019a), acupuncture plus herbal medicine (Zhou et al., 2017), or acupuncture plus western drugs (Wang et al., 2020b) provide more beneficial effects for cognitive function in AD patients than western drugs alone. However, to our knowledge, the proposed mechanisms of action of acupuncture for AD have not been systematically reviewed and discussed. Here, we comprehensively summarize and review the current experimental evidence of the therapeutic effects of acupuncture for AD. Based on the findings, significant issues for future studies are then put forward.

## Effects of Acupuncture on the Pathogenesis and Pathological Process of Alzheimer's Disease

### Aβ Metabolism

**Figure 1:**
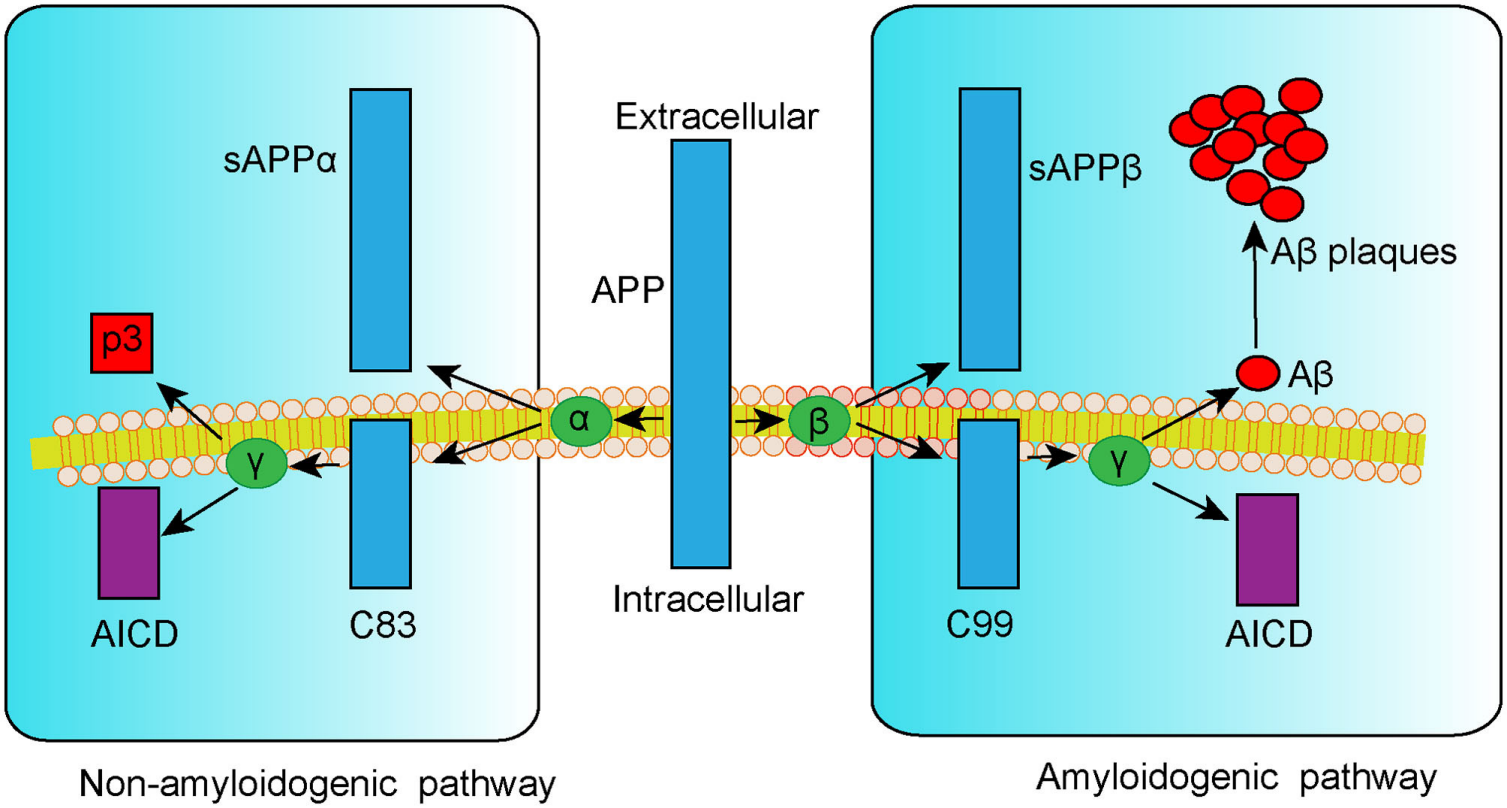
Schematic representation of APP metabolism via non-amyloidogenic and amyloidogenic pathways.

Extracellular senile plaque (SP) deposition due to dysregulated amyloid-β (Aβ) metabolism is a typical pathological change associated with AD. Aβ is a small peptide fragment formed by proteolytic cleavage of amyloid precursor protein (APP), a transmembrane protein that can be cleaved in a non-amyloidogenic or amyloidogenic pathway (Vassar et al., 1999). In the non-amyloidogenic pathway, APP is catabolized by α-secretase to the APP-α precursor (sAPPα) and the C83 α-subunit (C83), followed by the p3 fragment and APP intracellular domain (AICD) cleaved by γ-secretase. By contrast, in the amyloidogenic pathway, APP is first cleaved by β-secretase 1 (BACE1) into the APP-β precursor (sAPPβ) and C99 β-subunit fraction (C99). Additional processing of C99 by γ-secretase leads to the generation of either Aβ_1−40_ or Aβ_1−42_ peptides, which are considered to be responsible for the formation of toxic SP (Figure 1). According to the amyloid cascade hypothesis of AD, the formation, aggregation, and deposition of Aβ peptides result in a series of pathogenic processes including neuroinflammation, mitochondria damage, neuron apoptosis, and tau hyperphosphorylation. These events can, in turn, aggravate Aβ deposition and result in a vicious cycle, triggering cascade amplification effects and ultimately leading to neurodegeneration (Hardy and Higgins, 1992; Selkoe and Hardy, 2016). Thus, targeting Aβ formation and clearance is a potential therapeutic approach for treating AD.

Accumulating evidence supports that acupuncture can decrease Aβ deposition in AD patients and in AD-like animal models. Jiang et al. (2019) reported that acupuncture alone or acupuncture combined with donepezil treatment reduced cortical Aβ amyloid content and improved spatial learning and memory in SAMP8 mice. In addition, EA was shown to decrease the hippocampal Aβ plaque load in APP/PS1 mice via downregulation of APP and BACE1 levels to alleviate cognitive deficits (Yang et al., 2018; Tang et al., 2019), while inhibition of the JNK signal transduction pathway was implicated in EA-induced APP downregulation (Tang et al., 2020). Zhang et al. (2017) reported that activation of the peroxisome proliferator-activated receptor γ (PPAR-γ) by EA treatment contributed to the reduced hippocampal Aβ burden in Aβ_1−40_-induced AD rats. It was suggested that activation of PPAR-γ can reduce Aβ generation via inhibition of BACE1 (Wang X. et al., 2017). PPAR-γ activation can also reduce the number of activated microglia, resulting in lower release of pro-inflammatory cytokines (Heneka et al., 2005). Thus, PPAR-γ is a potential therapeutic target for AD (Khan et al., 2019). Several kinases related to Aβ clearance are also involved in EA-induced decreases in Aβ deposition, namely, ApoE, lipoprotein lipase (Tang et al., 2018), insulin-degrading enzyme (Yang et al., 2018), and neprilysin (Jha et al., 2015; Wang X. et al., 2018). Taken together, these findings support that acupuncture reduces Aβ deposition via effects on cleavage and degradation pathways.

The existing studies investigating the effects of acupuncture on Aβ have mainly focused on whether acupuncture reduced Aβ plaque or the effects of acupuncture on molecular proteins involved in the amyloidogenic pathway. Fewer studies have examined effects on the pathological process resulting from β-amyloid accumulation and deposition, which could be a more convincing mechanism for explaining acupuncture-induced neuroprotection. Besides, the investigated pathways associated with acupuncture-induced decreases in Aβ load are not in-depth. Additional signaling pathways involved in the cleavage and degradation of Aβ, as well as the interaction with Aβ, warrant further research attention.

### Tau Phosphorylation

**Figure 2:**
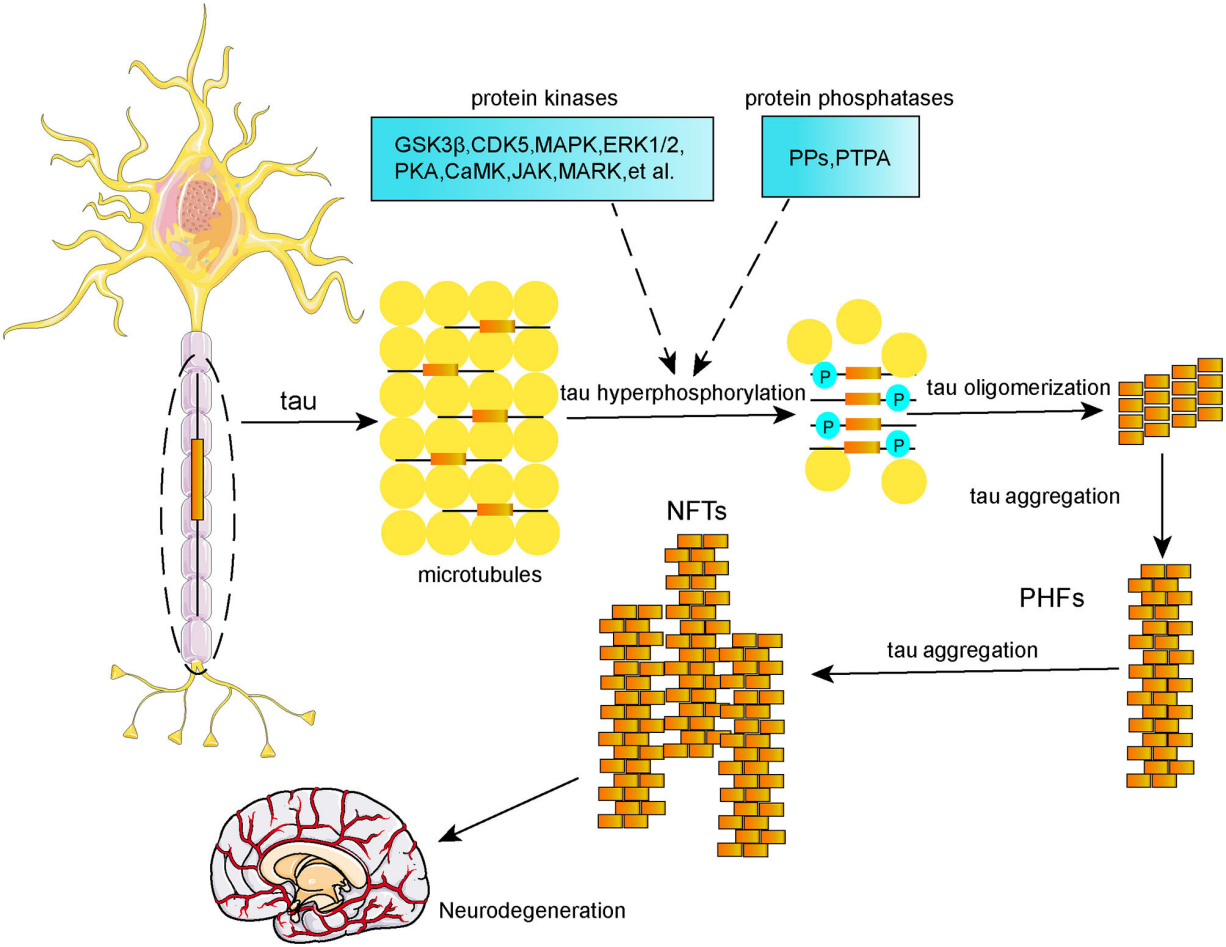
Schematic representation of tau phosphorylation and formation of NFTs.

Tau protein is a microtubule-associated protein (MAP) that is highly enriched in neurons located in frontal, temporal, hippocampal, and entorhinal regions. Its main biological function is to promote microtubule assembly and stabilize microtubules, which are significant for normal axonal transport and synaptic plasticity (Šimić et al., 2016). Tau protein undergoes various posttranslational modifications, including phosphorylation, acetylation, methylation, ubiquitination, SUMOylation, nitration, glycosylation, truncation, and splicing. Tau phosphorylation is the main posttranslational modification event (D'Souza and Schellenberg, 2005). Normal tau phosphorylation plays a significant role in hippocampal neurogenesis (Hong et al., 2010) and anti-apoptosis (Li et al., 2007); however, tau hyperphosphorylation can reduce its affinity for microtubules, and further results in a decreased microtubule stability and disordered axoplasmic transport, which subsequently affect the synthesis, transport, release, and uptake processing of neurotransmitters, thereby leading to neurodegeneration (Spillantini and Goedert, 2013). It is well-established that imbalanced regulation of tau phosphorylation and dephosphorylation results in tau hyperphosphorylation due to dysregulation of protein kinases and protein phosphatases (Figure 2). These protein kinases and protein phosphatases include glycogen synthase kinase (GSK-3β), cyclin-dependent kinase 5 (CDK5), janus kinase (JAK), mitogen-activated protein kinase (MAPK), extracellular signal-regulated protein kinases 1 and 2 (ERK1/2), protein kinase A (PKA), calmodulin kinase II (CaMKII), microtubule affinity-regulating kinase (MARK), protein phosphatase type 2A (PP2A), among others. Neurofibrillary tangles (NFTs), which are composed of hyperphosphorylated tau in the form of paired helical filaments (PHFs), are a hallmark of the AD brain, and the formation of NFTs is positively correlated with the degree of dementia (Berg et al., 1998; Giannakopoulos et al., 2003) rather than Aβ plaques (Thal et al., 2002; Braak et al., 2011). Thereby, tau hyperphosphorylation-based therapies could be a promising strategy for AD.

Yang et al. reported that 2 Hz EA treatment at GV20, BL23, and GV14 reduced hippocampal phosphorylation levels at the Ser202 and Thr231 sites of tau and decreased tau mRNA expression in SAMP8 mice. Behaviorally, these mice showed improvements in learning and memory ability in the Morris water maze test (Yang et al., 2020). Wang et al. reported that EA significantly reduced phosphorylation levels at the Ser199 and Ser202 epitopes via inhibition of CDK5 activity in Aβ_25−35_-induced AD rats (Wang et al., 2020a). In addition, EA at a low burst frequency of 2 Hz decreased phosphorylation levels at the Ser396/404, Ser202, and Ser262 epitopes in the cortex and hippocampus of streptozotocin-induced diabetic rats. Inhibition of GSK-3β and p38 by EA is involved in the counteraction of streptozotocin-induced increases in tau phosphorylation (Rocco et al., 2013). As the main protein kinase associated with tau phosphorylation, GSK-3β is able to phosphorylate a number of phospho-tau epitopes including Thr205, Thr231, Ser396, Ser404, Ser202, Ser262, and Ser214 (Maqbool et al., 2016). Furthermore, Zhang et al. (2017) demonstrated that EA decreased tau phosphorylation levels via inhibition of the p38 MAPK signaling pathway. Mounting evidence supports a correlation of the p38 MAPK signaling pathway with both Aβ deposition (Kheiri et al., 2018) and tau phosphorylation (Sheng et al., 2001; Feijoo et al., 2005; Munoz and Ammit, 2010).

The ability of acupuncture to alter the activity of other protein kinases to influence tau phosphorylation levels in AD requires further research. Of the protein phosphatases implicated in tau dephosphorylation and aggregation, PP2A plays the largest role (Martin et al., 2013). Thus, the effects of acupuncture on protein phosphatases that are able to dephosphorylate tau protein should be investigated. Factors besides protein kinases and protein phosphatases are also known to promote tau hyperphosphorylation, including other posttranslational modifications. It has been demonstrated that SUMOylation of tau at K340 promotes tau phosphorylation at multiple sites (Luo et al., 2014), and O-glycosylation attenuates tau phosphorylation (Li et al., 2006), whereas N-glycosylation aggravates tau phosphorylation and accumulation (Wang et al., 1996). Future studies should aim to expand our understanding of how acupuncture affects other posttranslational modifications of tau protein, which in turn affect tau phosphorylation.

### Neurotransmitters

**Figure 3:**
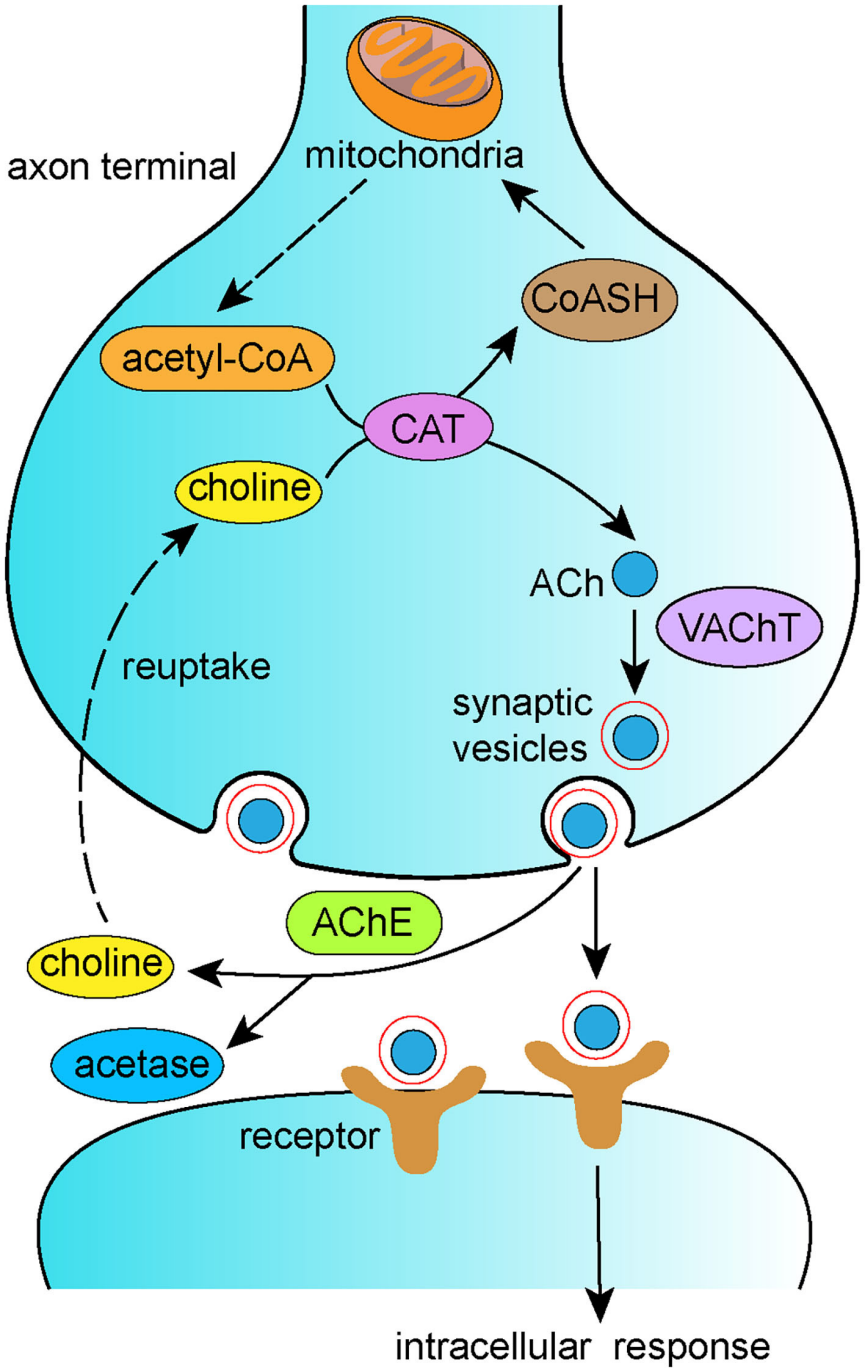
Schematic representation of synthesis of ACh and cholinergic transmission.

According to the cholinergic hypothesis, the cholinergic system plays a significant role in the pathogenesis of AD. The cholinergic system is involved in primary physiological processes such as attention, learning, memory, sleep, and stress response (Hasselmo et al., 1992; Bucci et al., 1998; Miranda and Bermúdez-Rattoni, 1999). Loss of cholinergic function is associated with decreased synthesis of acetylcholine (ACh) in the basal forebrain, which contributes to memory loss in AD (Whitehouse et al., 1981). In the cytoplasm of cholinergic neurons, choline and acetyl-coenzyme A (acetyl-CoA) are synthesized into ACh by choline acetyltransferase (ChAT). ACh is then transported from the cytoplasm into synaptic vesicles via the vesicular acetylcholine transporter (VAChT) and hydrolyzed by acetylcholinesterase (AChE) in the synaptic cleft into choline, which is eventually reuptaken into presynaptic cholinergic neurons (Figure 3). Disordered regulation of the synthesis, storage, transportation, or degradation of ACh can all result in cognitive dysfunction (Ferreira-Vieira et al., 2016). It is well-recognized that reduced CAT contributes to behavioral dysfunction, and reduced CAT has been reported in the hippocampal and neocortical regions of AD brains. Thus, ACh supplementation therapy has been proposed as a treatment for AD. Accumulating research has reported that acupuncture alters ACh levels via modulation of its metabolism. Yun et al. (2017) reported that laser acupuncture reversed post-ischemic decreases in ChAT in the hippocampal CA1 region and attenuated cognitive impairment in middle cerebral artery occlusion rats. In addition, EA has been shown to counteract LPS-induced decreases in α7nAChR, ACh content, and ChAT activity and to prevent LPS-induced increases in AChE activity, thus improving both working and spatial memory (Han et al., 2018). Lee et al. (2014) found that acupuncture stimulation at GV20 improved scopolamine-induced cognitive deficits via activation of the cholinergic system, as evidenced by increased levels of ChAT, choline transporter 1, and VAChT.

Glutamate (Glu), the most abundant excitatory neurotransmitter in the central nervous system (CNS), plays significant roles in modulating synaptic transmission, neuronal survival and differentiation, synaptic plasticity, learning, and memory (Benarroch, 2018). Glu receptors exist in the form of G-protein-coupled receptors (GPCR, also termed metabotropic receptors) and ionotropic receptors, such as the N-methyl-D-aspartate receptor (NMDAR) and α-amino-3-hydroxy-5-methyl-4-isoxazolepropionic acid receptor (AMPA receptor) (Wang and Reddy, 2017). Glu mostly binds to ionotropic NMDAR receptors to modulate calcium and sodium influx into neurons. Overaction of Glu due to disordered reuptake can result in excessive calcium influx within neurons, leading to dysfunctional synaptic transmission, neuron damage, and neurodegeneration (Hynd et al., 2004). Clinical evidence suggests that memantine, an NMDA receptor antagonist, can hinder AD progression (Parsons et al., 2007). Lin et al. (2018) reported that EA stimulation at an alternating burst frequency of 1 and 20 Hz alleviated cognitive dysfunction in APP/PS1 transgenic mice. Enhanced hippocampal Glu metabolism, as measured by magnetic resonance spectroscopy, and an increased number of surviving neurons were also observed, implying that EA ameliorated memory impairment via reduction in Glu content. Similarly, 2 Hz EA was demonstrated to improve learning and memory ability in vascular dementia rats by inhibiting Glu-NMDAR-mediated excitotoxity (Zhang et al., 2016).

Additional neurotransmitters to those described above have been implicated in synaptic plasticity and neuroinflammation in AD, including serotonin, noradrenaline, dopamine, and GABA. Pathological changes in monoaminergic nuclei, particularly the serotonergic dorsal raphe nucleus, noradrenergic locus coeruleus, and dopaminergic nuclei, have been observed during the early course of AD and are thought to influence symptoms and pathogenesis (Šimić et al., 2017). However, few studies have investigated the effects of acupuncture on these neurotransmitters in AD animal models. Since alterations in monoaminergic systems appear to play a significant role in AD, neurotropic virus-mediated neural circuit tracing technology and chemogenetic techniques could be used to explore the effects of acupuncture on monoaminergic systems and their interactions with the hippocampus, cholinergic system, or prefrontal cortex.

### Neurogenesis

In various AD mouse models, it has been shown that adult hippocampal neurogenesis is impaired (Zeng et al., 2016; Richetin et al., 2017; Zaletel et al., 2018). Hippocampal neurogenesis is also decreased in human AD patients, but abundant in the dentate gyrus of neurologically healthy subjects (Moreno-Jiménez et al., 2019). In a recent study, Tobin et al. (2019) confirmed that hippocampal neurogenesis occurs in both aging adults and AD patients. Thus, promoting adult hippocampal neurogenesis could be a therapeutic strategy for AD (Mu and Gage, 2011). Tang et al. (2006) reported that acupuncture at GV20, KI1, KI3, and SP10 not only upregulated ChAT activity in the medial septum but also increased nerve growth factor (NGF) levels in the hippocampal CA3 area in AD model rats. NGF plays critical roles in cell survival and is implicated in memory deficits in AD (Iulita and Cuello, 2014). It has been proven that NGF can prevent cholinergic neuron degeneration. Interestingly, it has been suggested that exogenous NGF supplementation enhances the APP nonamyloidogenic cleavage pathway and reduces the Aβ burden in the APP/PS1 mice brain (Yang et al., 2014). Furthermore, biodelivery of NGF to the basal forebrain has been shown to reduce brain atrophy in AD patients (Ferreira et al., 2015). Thus, it is reasonable to assume that increasing NGF content could be a treatment for AD. There is evidence that acupuncture can increase NGF levels and alleviate cognitive dysfunction in animal models of cerebral ischemia (Chen et al., 2015; Ding et al., 2017; Zhao J. et al., 2019). Rocco et al. (2013) also reported that EA counteracted diabetes-associated tau hyperphosphorylation and decreases in NGF and ChAT.

Brain-derived neurotrophic factor (BDNF), part of the neurotrophic factor family, plays important roles in modulating neuronal differentiation, proliferation, nutrition, and synaptic plasticity (Kowiański et al., 2018; Numakawa et al., 2018). BDNF activates tyrosine receptor kinase (TrkB), which then stimulates several intracellular signaling cascades including the MAPK/ERK, PLCγ, and PI3K/Akt signaling pathways (Mohammadi et al., 2018). As BDNF has been implicated in AD pathology, BDNF-based therapy could be a promising strategy for treating AD (Lu et al., 2013; Song et al., 2015). Choi et al. (2018) showed that the induction of adult hippocampal neurogenesis combined with elevation of BDNF levels ameliorated cognitive impairments in 5 × FAD mice. Acupuncture-induced rescue of cognitive dysfunction in AD mice is associated with elevation of BDNF levels (Li et al., 2014; Lin et al., 2016, 2018). Acupuncture has also been reported to promote the proliferation and differentiation of neural stem cells (NSCs) in the hippocampus of SAMP8 mice following NSCs transplantation treatment. Furthermore, it was demonstrated that acupuncture upregulated the expression of hippocampal cytokines involved in NSC proliferation and differentiation, including BDNF, basic fibroblast growth factor, and epidermal growth factor, thereby promoting the repair of injured neurons and improving cognitive function (Zhao et al., 2017).

At present, a few studies have examined the effects of acupuncture on neurogenesis in AD and the underlying mechanism. Multiple extrinsic and intrinsic factors that are involved in regulating neurogenesis are also altered in AD. These extrinsic modulators include metabolic growth factors, such as VEFG (Wittko et al., 2009), BDNF, IGF-1 (Yuan et al., 2015), FGF-2 (Woodbury and Ikezu, 2014), and IGF, that contribute to the proliferation, maturation, and migration of NSCs. The intrinsic modulators include Wnt signaling (Lie et al., 2005), Notch signaling (Imayoshi et al., 2010), Sonic hedgehog signaling (Lai et al., 2003), and epigenetic modifications (Li X. et al., 2016). However, whether acupuncture can regulate these modulators to promote neurogenesis in AD remains unknown. Studies of the effects of acupuncture on the extrinsic and intrinsic factors mentioned above could provide further evidence of how acupuncture alters neurogenesis in AD.

### Synapse and Neuron Damage

It is well-established that synapse and neuron loss are strongly correlated with cognitive dysfunction in AD, suggesting a causal role of compromised synaptic and neuronal integrity in AD pathogenesis. In addition, AD animal models show deficits in both synaptic morphological plasticity and synaptic transmission (Guo et al., 2017; Chakroborty et al., 2019). Accumulating evidence has demonstrated that acupuncture may ameliorate synapse and neuron damage caused by ischemic stroke (Jittiwat, 2019), inflammation, oxidative stress (Du et al., 2018), and neurodegeneration (Zhao Y. et al., 2019). Li et al. (2012) reported that acupuncture reduced neuron loss in the hippocampal CA3 and DG areas in SAMP8 mice and attenuated memory impairments. In addition, acupuncture increased the number and total length of apical and basal dendritic branches in the hippocampal CA1 region in the mouse model (Kan et al., 2018). EA stimulation at an alternating frequency of 2 and 15 Hz was found to attenuate neuronal injury in Aβ_1−42_-induced AD rats. Interestingly, this effect was accompanied by reduced levels of reactive oxygen species (ROS), malondialdehyde (MDA), and 8-OH-dG and increased total antioxidant capacity (T-AOC), suggesting a correlation between EA-induced neuron protection and anti-oxidative stress. Further study demonstrated that inhibition of NOX2-related oxidative stress, as evidenced by decreased NOX2 expression, contributed to the EA-induced neuroprotective effects (Wu et al., 2017). Huang et al. (2018) reported that 2-Hz EA ameliorated hippocampal neuron injury and improved spatial learning and memory impairments in AD rats via activation of the SIRT1/PGC-α pathway to counteract oxidative stress damage. Also, it was suggested that EA's inhibitory effect on GSK-3β activity (Yu et al., 2018) and the AMPK/eEF2K/eEF2 signaling pathway (Dong W. et al., 2019) attenuated synaptic ultrastructure damage, thereby restoring cognitive function in AD animals. Yu et al. reported that high-frequency EA at 50 Hz, rather than low- or medium-frequency EA, exerted stronger protective effect on synapses (Yu et al., 2018), highlighting the need for further studies of the optimal EA stimulation dose. Our recent findings (Yu et al., 2020) showed that EA alleviated memory deficits, attenuated dendritic spine loss, and rescued neuronal microtubule damage in the hippocampal CA1 area of aging rats, likely via inhibition of the GSK3β/mTOR signaling pathway.

Several studies have found that EA enhances synaptic transmission. Shen et al. (2010) reported that EA at GV20, GV14, BL23, and LI3 enhanced hippocampal long-term potentiation (LTP), the most prominent cellular model of memory formation, in Aβ_25−35_-induced AD rats. In addition, 2 Hz EA at ST36 and SP6 enhanced LTP of perforant path-DG granule neurons (He et al., 2012). Although these findings suggest that EA can enhance synaptic transmission in AD animals, the molecules and pathways involved in this neuroprotective effect remain unclear. Notably, acupuncture was found to enhance LTP in the hippocampus by increasing norepinephrine levels and activating β1-adrenergic receptors in a vascular dementia animal model (Xiao et al., 2018). As the noradrenergic system is also implicated in AD pathogenesis (Feinstein et al., 2016; Jeon et al., 2018), it is possible that acupuncture prevents synaptic transmission impairment via the modulation of noradrenergic system pathways.

### Autophagy

Autophagy is an essential lysosomal degradation pathway in which misfolded or aggregated proteins and damaged organelles are cleared from the intracellular space (Lee et al., 2013). Autophagy acts in the mammalian target of rapamycin (mTOR)-dependent pathway or mTOR-independent pathway to maintain cellular homeostasis. Accumulating evidence has implicated dysfunctional autophagy in the pathogenesis of neurodegenerative diseases such as AD, Parkinson's disease, and amyotrophic lateral sclerosis (Menzies et al., 2017). By mediating degradation and clearance of Aβ and tau, autophagy plays a neuroprotective role in AD. Alteration of the PI3K/Akt/mTOR signaling pathway, one of the mTOR-dependent autophagy pathways, has been reported at the early stages of AD together with increased Aβ_1−42_ levels and reduced LC3II and Beclin-1 (Tramutola et al., 2015). Microtubule-associated protein 1 light chain 3 (LC3) and Beclin-1 are vital for phagophore elongation and autophagosome biogenesis (Kraft and Martens, 2012; Bernard and Klionsky, 2014). Conversion from a nonlipidated form (LC3 I) to a phosphatidylethanolamine-conjugated form (LC3 II) is necessary for the formation of complete and functional autophagosomes. An elevated LC3 II/LC3 I ratio indicates enhanced autophagy activity (Kraft and Martens, 2012).

Increased autophagic vacuoles containing Aβ_1−40_, Aβ_1−42_, and APP have been observed in AD brains (Yu et al., 2004, 2005). Therefore, targeting autophagy modulators may be an effective AD treatment. Xue et al. (2014) found that EA at GV20 and KI1 decreased cortical Aβ_1−42_ levels in AD mice, which was associated with enhanced autophagy activity as demonstrated by elevated autophagosomes after EA treatment. In addition, EA at GV20 and BL23 increased the autophagy-related protein Beclin-1 level and LC3 II/LC3 I ratio, but decreased Aβ plaque and neuronal apoptosis in the hippocampal CA1 region (Guo et al., 2016), suggesting that modulation of autophagy modulators by EA is involved in the rescue of cognitive dysfunction in AD model animals.

However, a few studies have investigated the effects of acupuncture on specific autophagic pathways, such as mTOR-dependent or mTOR-independent signaling pathways. Previous studies have indicated that acupuncture can modulate autophagy via several pathways including the mTOR-independent autophagy lysosome pathway (Tian et al., 2016) and AMPK-dependent pathway (Zeng et al., 2018) in Parkinson's disease and myocardial infarction injury. Thus, the pathways and molecules involved in acupuncture-induced changes in autophagy in AD warrant specific study. Of the studies that have examined these topics, all have reported that acupuncture enhanced autophagy activity in AD and promoted clearance of mutant or misfolded proteins. These “positive” results should not be misunderstood to mean that stimulation of autophagy by acupuncture is purely neuroprotective in AD. Notably, excessive autophagy could be detrimental to neurons with underlying dysfunctional proteostasis, since it is still debated whether accumulation of uncleared autophagosomes may be a cause or the consequence of the dysfunction of autophagy induction pathways. By contrast, “negative results” of acupuncture on autophagy activity and the ultimate effects on pathologies and cognition deficits in AD have received little research attention. Thus, the effects of acupuncture with varying stimulus parameters on the promotion or inhibition of autophagy in AD should be examined and compared in future studies.

### Apoptosis

Apoptosis refers to the process of programmed cell death, which is distinct from necrosis (Kennedy, 2015). Normal apoptosis plays a significant role in self-renewal and the maintenance of homeostasis, whereas hyperactive neuronal apoptosis can result in neurodegenerative diseases such as AD and Parkinson's disease (Radi et al., 2014). Aβ deposition, NFTs, neuroinflammation, and oxidative stress in the AD brain can all result in neuronal apoptosis, which may further aggravate AD pathology (Radi et al., 2014). Increased apoptosis-associated markers have been observed in AD brains (Anderson et al., 1996). Several studies have shown that EA can suppress hippocampal neuron apoptosis by acting on apoptosis-associated proteins, including Bcl-2, Bax, Caspase-3, and Caspase-9 (Li X. y. et al., 2016; Huang R. et al., 2019; Zhang et al., 2019). Guo et al. (2015) reported that EA at GV20 and BL23 reduced neuronal apoptosis and induced downregulation of Notch1 and Hes1 mRNA in the hippocampus of Aβ_1−42_-induced AD rats, implicating inhibition of the Notch signaling pathway in the anti-apoptotic effects of EA. The anti-apoptotic effects of EA have been reported in other neurological disease. Liu et al. (2016) showed that EA suppressed apoptosis by inhibiting autophagosome formation and autophagy activity via the mTORC1–ULK complex–Beclin1 pathway in ischemic stroke model animal. Activation of the PI3K/Akt-ERK signaling pathway is also strongly correlated with anti-apoptosis mediated by acupuncture in spinal cord injury (Renfu et al., 2014). Additional specific signaling pathways involved in the anti-apoptotic effect of acupuncture in AD require validation in future studies.

### Neuroinflammation

The critical role of neuroinflammation in the pathogenesis of AD has been extensively discussed in previous reviews (Heneka et al., 2015; Calsolaro and Edison, 2016; Ransohoff, 2016). Inflammatory cytokines overexpressed in proximity to Aβ plaques and NFTs are known to promote the production of Aβ peptides (Tuppo and Arias, 2005). These inflammatory cytokines, such as interleukin-1β (IL-1β), tumor necrosis factor-α (TNF-α), and interleukin-6 (IL-6), aggravate neuroinflammation via deposition of Aβ plaques and thus exert neurotoxic effects (Belkhelfa et al., 2014). Activation of neuroinflammation and immune pathways is closely related to abnormal levels of pro-inflammatory cytokines in the cerebrospinal fluid and blood in AD (Swardfager et al., 2010; Rubio-Perez and Morillas-Ruiz, 2012; Brosseron et al., 2014; Liu et al., 2014). Activated glia surrounding Aβ plaque is considered a hallmark of neuroinflammation. Chronic and sustained glial activation, as well as pro-inflammatory cytokine release, can lead to neurodegeneration and cognitive deficits (Hoozemans et al., 2006). However, whether neuroinflammation is a cause or consequence of AD remains under debate, as microglia and astrocyte activation and Aβ deposition are strongly correlated with cognitive dysfunction in AD (Heneka et al., 2013; Calsolaro and Edison, 2016). Thus, attenuation of neuroinflammation could be a promising AD treatment.

Acupuncture has been shown to yield anti-inflammatory effects in various diseases such as pain (Gao et al., 2018), diabetes (Huang et al., 2019b), ischemic stroke (Ma et al., 2019), and myocardial ischemia (Wang J. et al., 2018). In recent years, several studies have reported that acupuncture can also alleviate inflammation in AD. Li et al. (2019) found that acupuncture improved cognitive function and attenuated inflammation in SAMP8 mice via inhibition of the PI3K/PDK1/Npkc/Rac1 signaling pathway. Downregulation of the JAK/STAT3 pathway was also found to contribute to EA-induced anti-inflammatory effects in Aβ_1−42_ induced AD rats (Liu et al., 2019). Mounting evidence supports the role of NLRP3 inflammasome activation in mediating neuroinflammation (Heneka et al., 2013), and inhibition of NLRP3 inflammasome-related proteins can restore cognitive function in AD (Dempsey et al., 2017; Wang D. et al., 2017; Feng et al., 2018).

Several studies have reported that acupuncture ameliorated hippocampal neuroinflammation via downregulation of the NLRP3 inflammasome and decreased production of downstream pro-inflammatory cytokines like IL-1β and Caspase-1 (Jiang et al., 2018; Ding et al., 2019), thus improving learning and memory abilities in SAMP8 mice. Furthermore, EA-induced inhibition of NLRP3 inflammasome activation via CB2 receptors has been shown to relieve inflammatory pain (Gao et al., 2018). Acupuncture also has inhibitory effects on glia activation. Zhang et al. (2013) reported that acupuncture prevented neuron loss and decreased the number of activated astrocytes in the hippocampal CA1 and CA3 regions of SAMP8 mice. Cai et al. (2019) demonstrated that EA stimulation ameliorated cognitive impairment via inhibition of synaptic degeneration and neuroinflammation in 5xFAD mice, as evidenced by decreased expression of CD11b (for microglia) and GFAP (for astrocytes) in the prefrontal cortex. Similarly, EA was reported to attenuate microglia-mediated Aβ_1−42_ deposition in the prefrontal cortex, as supported by a reduction in colocalized Aβ_1−42_ and CD68 (a microglia marker). In addition, EA can attenuate reference memory deficits in APP/PS1 transgenic mice, likely via inhibition of the astrocytic N-myc downstream-regulated gene 2 (Wang et al., 2014). Taken together, these findings support that acupuncture can attenuate neuroinflammation and rescue cognitive impairments in AD animal models. However, these studies have focused specifically on the CNS, and few studies have investigated the effects of acupuncture on systemic inflammation. Notably, systemic inflammation can have downstream effects on brain function via neuro-immune communication (Cao and Zheng, 2018). For example, a recent study demonstrated that EA activated distinct sympathetic pathways and modulated systemic inflammation in a somatotopic manner in a lipopolysaccharide (LPS)-induced inflammatory model that can be considered an AD-like inflammatory model (Liu et al., 2020). Circulating inflammatory proteins outside of the CNS can increase inflammatory signaling within the CNS, promoting activation of astrocytes and microglia and thus neurodegeneration (Walker et al., 2019). In light of these findings, it would be interesting for future studies to investigate the effects of acupuncture on peripheral-central neuroimmune communication in AD.

### Glucose Metabolism

Diabetes is known to be a significant risk factor for AD (Barnes and Yaffe, 2011; Silva et al., 2019), and AD can be considered type 3 diabetes mellitus (Leszek et al., 2017). Hippocampal insulin resistance is commonly observed in both AD patients (Talbot et al., 2012) and AD model animals (Velazquez et al., 2017). Accumulating evidence supports alterations in glucose metabolism and blood flow in cognition-related brain regions in AD patients (Nishimura et al., 2007; Dukart et al., 2013; Zilberter and Zilberter, 2017). Decreased glucose metabolism in the hippocampus, precuneus, and cingulate gyrus appear to be closely related to the severity of cognitive impairment (Roy et al., 2014). Dysregulated glucometabolism has also been observed in the hippocampus, hypothalamus, insular cortex, and striatum of AD rats (Lu et al., 2016). Identifying and assessing changes in CNS glucose metabolism may be a potential strategy for early and accurate diagnosis of AD (Teune et al., 2014; Kato et al., 2016; Oh et al., 2016; Takahashi et al., 2017). Furthermore, reversal of low cerebral glucose metabolic activity and insulin resistance has been shown to restore learning and memory in an AD mouse model (Kang et al., 2017; Nakamura et al., 2017; Walker et al., 2017).

It is recognized that acupuncture can regulate metabolic processes via effects on the neuroendocrine system (Yu et al., 2013; Ding et al., 2014). Mounting evidence supports the role of acupuncture treatment in increasing glucose metabolism and alleviating insulin resistance. Dong et al. (2015b) found that EA stimulation at the BL23 and GV14 acupoints enhanced brain glucose metabolism and increased ATP production, likely via activation of the SIRT1/PGC-1α pathway. Activation of the SIRT1/PGC-1α pathway can enhance mitochondrial oxidative function, which is significant for the maintenance of intracellular metabolic homeostasis (Fang et al., 2018; Fanibunda et al., 2019). EA has also been found to improve insulin sensitivity in diabetic animal models through activation of the SIRT1/PGC-1α (Liang et al., 2011) and SIRT1/FOXO1 pathways (Shu et al., 2020). Decreased activity of triose phosphate isomerase (TPI), a key enzyme in glucose metabolism, may result in abnormal accumulation of dihydroxyacetone phosphate (DHAP), thereby inhibiting the glycolysis process (Park et al., 2010). Glycometabolism disorder resulting from abnormal TPI activity is associated with learning and memory impairment (Tajes et al., 2013). Zhao et al. (2013) reported that EA improved cognitive impairment in SAMP8 mice by upregulating TPI activity and correcting abnormal glycolysis in the hippocampus. In addition, EA-induced improvement in cognition function in SAMP8 mice is associated with activation of AMPK (Dong et al., 2015a), a vital signal in regulating glucose and lipid metabolism (Misra, 2008). Activation of AMPK has been shown to improve altered metabolism in the CNS as well as learning and memory in AD model animals (Dong Y. et al., 2019).

Liu et al. found that EA at GV20 increased glucose metabolism in several brain areas including the cortex, hippocampus, cingulate gyrus, basal forebrain septum, brain stem, and cerebellum in APP/PS1 transgenic mice. This finding further supports the activation of AMPK and AKT in EA-induced increases in cortical and hippocampal glucose metabolism (Liu et al., 2017). Using brain imaging technologies such as positron emission tomography (PET), it is now possible to visualize AD-induced changes in brain glucose metabolism and regional brain blood flow changes in an objective way. Cui et al. (2018) showed that acupuncture at the GV24 and GB13 acupoints improved learning and memory abilities in Aβ_1−42_-induced AD rats, possibly by increasing glycolysis metabolism in the thalamus, hypothalamus, and brain stem areas. Ding et al. (2019) found that acupuncture rescued cognitive dysfunction in SAMP8 mice by inhibiting the astrocytic phospholipase A2-arachidonic acid pathway, which resulted in increased blood flow in the prefrontal lobe and hippocampus. Increased glycolysis in the hippocampus after EA treatment was also observed in APP/PS1 transgenic mice (Cao et al., 2017). Furthermore, EA has been shown to improve glycolysis in several cognition-related brain regions including the limbic system (pyriform cortex), temporal lobe (olfactory cortex), amygdala, and hippocampus in AD-like pathology rats (Lu et al., 2014). Enhanced glucose metabolic activity in the hippocampus, thalamus, hypothalamus, and frontal/temporal lobes, accompanied by restored memory, following acupuncture treatment was observed in SAMP8 mice (Lai et al., 2016). Using microPET, 2 Hz EA stimulation was shown to increase glucose metabolism in the frontal cortex and hypothalamus in 5xFAD mice (Cai et al., 2019). In summary, these findings suggest that enhancement of glucose metabolism in cognition-related brain regions could be an important mechanism of the beneficial effects of acupuncture in AD. However, few studies have examined the underlying mechanisms of acupuncture-induced glucose metabolism increases in AD. Rescuing brain energy failure is seen as an emerging therapeutic approach for aging-related neurodegenerative disorders. The gut–brain axis, neuroendocrine crosstalk, interactions among neuronal loops, and mitochondrial function are all known to regulate brain energy metabolism and could be useful directions for future studies of the underlying mechanisms of therapeutic acupuncture for AD.

### Brain Response

In recent years, functional magnetic resonance imaging (fMRI) has been used to examine acupuncture-associated changes in brain activity. It is known that regional blood flow, oxygen consumption, and the blood oxyhemoglobin/deoxyhemoglobin ratio all change after increased neuronal activity. Thus, changes in regional blood flow measured by fMRI are thought to represent changes in integrated neuronal activity. fMRI measures regional increases or decreases in neuronal activity based on increases or decreases in the blood-oxygen-level-dependent (BOLD) signal contrast, an objective measure that provides high temporal and spatial resolution without the requirement for an exogenous contrast medium (Gusnard et al., 2001; Shmuel et al., 2006).

It has been demonstrated that fMRI brain responses to acupuncture stimulation vary when different acupoints are stimulated. In a trial investigating differences in fMRI brain responses to acupuncture between healthy subjects and AD patients, Fu et al. observed that both the frontal and temporal lobes were activated by EA at PC6 in normal subjects. In contrast, the frontal and temporal lobes, cingulate gyrus, and cerebellum were activated in AD patients (Fu et al., 2005), suggesting that EA induced wider responses in cognition-related regions in AD brains. Jia et al. (2015) reported that acupuncture at the KI3 acupoint activated cognition-related regions including the medial frontal gyrus, inferior temporal gyrus, and posterior cingulate, which were distinct from the findings of sham acupuncture stimulation. In addition, stimulation at the LI4 and LR3 acupoints induced extensive activation and deactivation in cognition-related regions, vision-related regions, sensorimotor-related areas, basal ganglia, and cerebellum in patients with AD or mild cognitive impairment (MCI), but not in healthy controls (Shan et al., 2018). However, other studies have reported inconsistent results. Zheng et al. confirmed that acupuncture at the LI4 and LR3 acupoints not only increased neuronal activity in the hippocampus and precentral gyrus, but also enhanced functional connectivity between these two regions in AD patients. In addition, correlation analysis indicated strong relationships between functional activity, connectivity, and clinical performance (Zheng et al., 2018). Liang et al. reported that default mode network (DMN) connectivity between the left cingulate gyrus and right inferior parietal lobule was significantly compromised in AD patients. Acupuncture at the LI4 and LR3 acupoints not only increased impaired DMN connectivity but also enhanced DMN connectivity among the left posterior cingulate cortex, right middle temporal gyrus, and right inferior parietal lobule. In addition, the acupuncture effect on the middle temporal gyrus was strongly correlated with the severity of cognitive impairment (Liang et al., 2014). Acupuncture at the Tiaoshen Yizhi acupoints, a combination of acupoints based on Chinese acupuncture theory comprising EX-HN1, EX-HN3, PC6, KI3, ST40, and LR3, improved cognitive function in patients with MCI by increasing functional connectivity among cognition-related brain areas including the insula, dorsolateral prefrontal cortex, hippocampus, thalamus, inferior parietal lobule, and anterior cingulate cortex (Tan et al., 2017). In a functional near-infrared spectroscopy (fNIRS) study investigating the longitudinal effects of acupuncture in MCI patients, increased functional connectivity in the prefrontal cortex induced by acupuncture contributed to improved cognitive function (Ghafoor et al., 2019).

In summary, the neuroimaging findings provide relatively objective evidence for the therapeutic effects of acupuncture for AD. As the responding brain areas are inconsistent among the studies, it can be concluded that acupuncture can induce a wide range of cognition-related brain responses in AD and increase functional connectivity. The differences in responding brain areas could be due to the various acupoints stimulated, acupuncture method (electrical or manual), EA stimulation parameters (frequency, current, or wave type), or heterogeneities among the included participants. The underlying mechanism of increased functional connectivity among the activated brain areas has not yet been fully explained. In the future, investigating the specific types of activated neurons, as well as the projections and innervations of the responding brain nuclei, may provide a clearer and more specific understanding of the brain responses induced by acupuncture in AD.

## Conclusion

**Figure 4:**
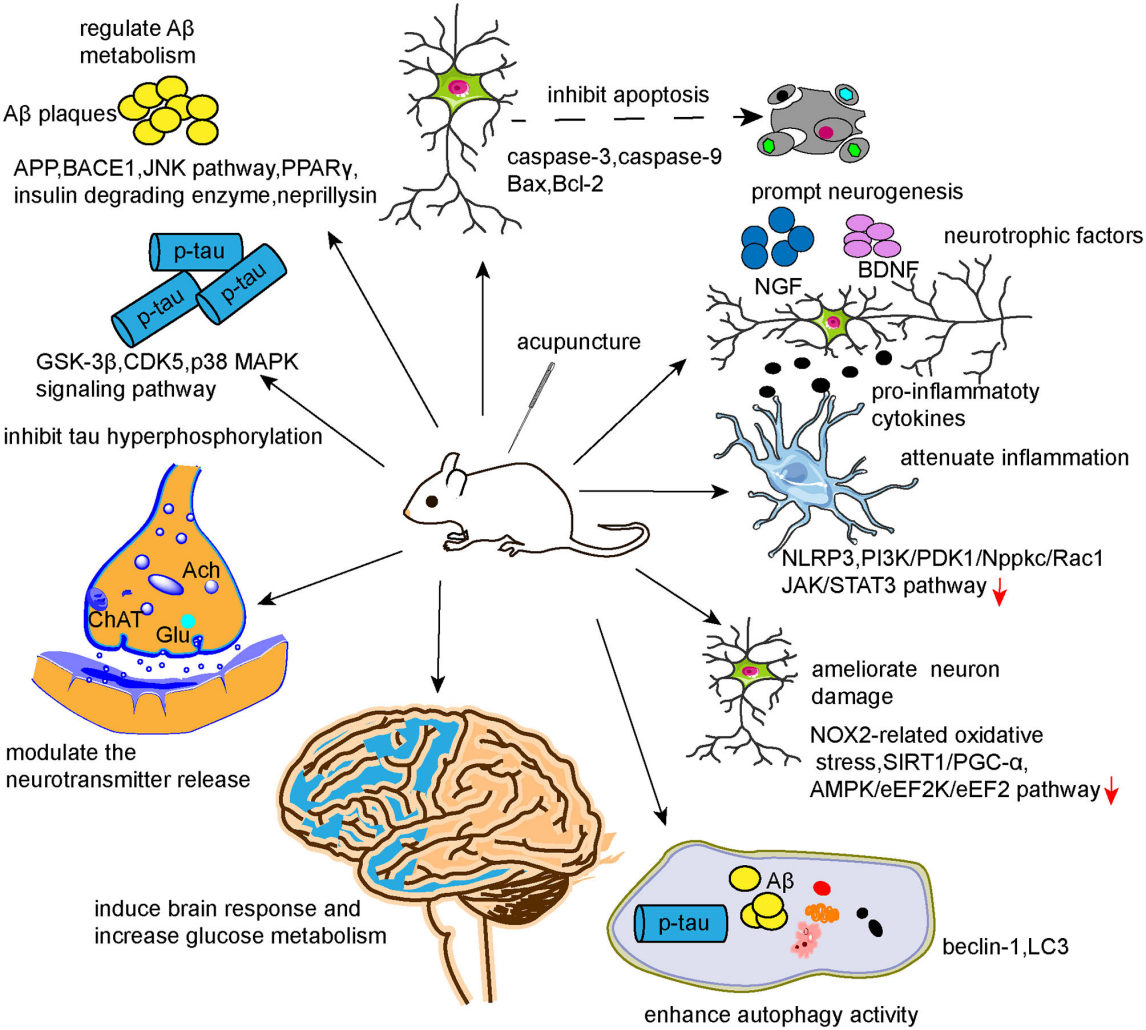
Mechanisms of action of acupuncture in AD.

From this updated review of the literature, we conclude that the underlying mechanisms of the beneficial effects of acupuncture in AD likely involve modulation of Aβ metabolism, tau phosphorylation, neurotransmitters, neurogenesis, synapse and neuron function, autophagy, neuronal apoptosis, neuroinflammation, cerebral glucose metabolism, and brain response. Together, these studies provide a base of scientific evidence to promote the clinical application of acupuncture as treatment for AD. However, several issues remain. First, as the pathogenesis of AD is complicated and remains unvalidated, the involved pathophysiologies are intertwined and may even coexist as a cause–consequence relationship. Previous studies investigating the mechanisms of acupuncture for AD mainly focused on a single factor, such as inflammation or dysregulated neurotransmitters, with less consideration of the links with other pathophysiologies or the impacts of parallel pathophysiologies, which may, to some degree, undermine the evidence. As illustrated in Figure 4, the effects of acupuncture can be achieved via multiple targets and pathways, which is in accordance with the features of systematic regulation by acupuncture. Therefore, it may be preferred to study the mechanisms of acupuncture in AD from a holistic view or systematic biology perspective. Multi-omics technologies, such as transcriptomics, proteomics, and metabolomics, could be adopted to explore the potential mechanisms of action of acupuncture in AD. Second, as acupuncture involves peripheral stimulation of sensitized points to regulate neural and visceral functions via multiple neural feedback systems, mapping the peripheral-neural circuits associated with AD using chemogenetic or optogenetic techniques could expand our understanding of the underlying mechanisms of acupuncture's benefits in AD. Third, the efficacy of acupuncture in AD cannot be validated based on the current evidence. Since AD is insidious, progressive, difficult to reverse, and had several nongenetic risk factors, the preventive effects of acupuncture for AD should be examined in future studies. Animal models that show AD-like pathologies caused by nongenetic risk factors, such as aging, diabetes, hypercholesterolemia, hyperhomocysteinemia, gastrointestinal microbiota, etc., could be adopted to study the preventive effects of acupuncture.

## Author Contributions

C-CY conceived the main ideas and wrote this paper. Y-JD designed the framework. S-QW helped search the references. LW helped illustrate the figures. Y-FL and L-HK helped revise the manuscript. All authors contributed to the article and approved the submitted version.

## Conflict of Interest

The authors declare that the research was conducted in the absence of any commercial or financial relationships that could be construed as a potential conflict of interest.
